# Needs of an uninsured equity-deserving minority patient cohort with physical disabilities during the first wave of the COVID-19 pandemic

**DOI:** 10.3389/fresc.2023.1000838

**Published:** 2023-02-17

**Authors:** D Claus, C Draganich, J Berliner, W Niehaus, J Berliner, D Magnusson, A. C. Smith

**Affiliations:** ^1^ Department of Physical Medicine and Rehabilitation, University of Colorado School of Medicine, Aurora, CO, United States; ^2^ Craig Hospital, Englewood, CO, United States; ^3^ School of Physical Therapy, Regis University, Denver, CO, United States

**Keywords:** access to health care, rehabilitation, minority health, spinal cord injuries, brain injuries, stroke, amputation, interdisciplinary/multidisciplinary

## Abstract

**Background:**

Patients with disabilities and those from diverse equity-deserving backgrounds have been disproportionately affected by the SARS COV-2 (“COVID-19”) pandemic.

**Objective:**

To describe the significant needs and social determinants of health that affected a group of uninsured patients (from equity-deserving groups) with rehabilitation diagnoses during the early months of the COVID-19 pandemic.

**Design:**

Retrospective cohort study utilizing a telephone-based needs assessment from April to October, 2020.

**Setting:**

Free interdisciplinary rehabilitation clinic serving patients with physical disabilities from equity-deserving minority backgrounds.

**Participants:**

51 uninsured, diverse patients with spinal cord injuries, brain injuries, amputations, strokes, and other diagnoses requiring interdisciplinary rehabilitation care.

**Methods:**

Using a non-structured approach, telephone-based needs assessments were collected monthly. Reported needs were summarized into themes and the frequencies of each theme were recorded.

**Results:**

From the total number of concerns, medical issues were reported with the highest frequency (46%), followed by equipment needs (30%) and mental health concerns (30%). Other frequently mentioned needs centered around themes of rent, employment, and supplies. Rent and employment were more frequently cited in earlier months, and equipment problems were more frequently cited in later months. A minority of patients reported they had no needs, some of whom had acquired insurance.

**Conclusions:**

Our objective was to describe the needs of a racially and ethnically diverse set of uninsured individuals with physical disabilities seen at a specialized interdisciplinary rehabilitation pro bono clinic during the early months of COVID-19. Medical issues, equipment needs, and mental health concerns were the top three needs. To optimally serve them, care providers must be aware of current and future needs for their underserved patients, especially if future lockdowns occur.

## Introduction

Individuals living with physical disabilities faced considerable hardship during the initial months of the SARS-CoV-2 (COVID-19) global pandemic (1, 2). During early discussions about the allocation of scarce healthcare resources, individuals with disabilities were often considered a lower priority (2) and were more likely to die within the first two months of the pandemic (1). In addition, lockdowns exacerbated mental health concerns (3, 4) and led to a reduction of family and healthcare support for those with physical disabilities (3, 5). As the pandemic continued, despite improved access and usage of telemedicine for the general population, this technology still presented barriers for persons with disabilities (6). Overall, people with disabilities were shown to have less access to healthcare, education, social services, food, and emergency supplies during the pandemic (4, 6).

Equity-deserving minority groups also experienced disparities during the COVID-19 pandemic (7). Especially vulnerable populations included those who were homeless, incarcerated, undocumented, or immigrants (8–10). Additionally, African American, Latinx, and Native American populations suffered disproportionately compared to the Caucasian population (11). During the first months of the pandemic, Hispanic and Latinx communities had higher rates of hospitalizations and death compared to Caucasians (11). Factors such as access to healthcare, immigration status, and language barriers contributed to the disparities seen in Latinx communities (11). Similarly, correlations demonstrated higher mortality from COVID-19 for African Americans and Asian Americans (12).

Given the existing literature, persons with a physical disability who also belong to equity-deserving ethnic or racial minority groups may have experienced exponentially greater difficulty, especially during the beginning of the COVID-19 pandemic (13). Even before COVID-19, a recent narrative review suggests that Hispanic patients may be at risk for worse outcomes after stroke, spinal cord injury (SCI), or traumatic brain injury (TBI), due to lack of access to rehabilitation and disparities regarding referral to specialized care (14). Similarly, another narrative review found that Black persons who had experienced a stroke, TBI, or SCI were less likely to receive care aligned with clinical guidelines (15).

Identifying specific needs of those patients with physical disabilities within the context of COVID-19 is necessary to inform public health recommendations and healthcare policy considerations that promote accessible participation in rehabilitation (16). It is especially important to describe the specific needs of individuals with physical disabilities who identify with an equity-deserving racial or ethnic minority group. In general, little is known regarding this population (13). There is a paucity of literature that reports specifically on the needs of those with physical disabilities from minority groups during the COVID-19 pandemic. This knowledge could be used to better equip healthcare systems in the event of other regional or worldwide health crises.

Accordingly, this manuscript aims to identify and describe the needs of a racially and ethnically diverse set of uninsured individuals with physical disabilities seen at a specialized interdisciplinary rehabilitation pro bono clinic located in Denver, Colorado, USA, during the first wave and lockdowns of COVID-19.

## Methods

This was a retrospective cohort study conducted at a local pro-bono clinic. This needs assessment was approved by the local institutional review board and conducted in accordance with the Declaration of Helsinki. Informed consent was obtained from all individual participants included in the study.

### Setting

Our specialized interdisciplinary rehabilitation pro-bono clinic serves equity-deserving uninsured individuals with catastrophic injury or physical disability (primarily from stroke, SCI, TBI, or amputations). The disciplines involved with our clinic include (but are not limited to) physiatrists, physical therapists, occupational therapists, speech language pathologists, social workers, pharmacists, rehab psychologists, adaptive equipment specialists, prosthetists, orthotists, nurses, and interpreters. Between April and June 2020, the clinic was closed due to the COVID-19 pandemic. To continue to serve patients, a virtual clinic was developed and telephone-based needs assessments were conducted for all patients previously served by the clinic. The calls served as an informal, monthly means of communicating with patients to assess their specific needs. When needs were identified, the clinic leadership attempted to assist in any way possible. These calls started in April 2020 and continued through November 2020.

### Participants

All patients previously referred to, or evaluated by, our clinic were eligible to be included. The majority of patients belong to equity-deserving ethnic and racial minority groups. Many patients are Spanish-speaking and from Latinx communities in the Denver metro area.

### Data collection

A team of volunteer patient navigators attempted to call all of the patients described above. Using a non-structured approach, the volunteers asked about a variety of topics related to health and healthcare, as well as social determinants of health (SDoH). Each patient was called once per month. The patients' answers were collected by multiple volunteers and stored in a secure online spreadsheet.

### Analysis

Patient reported needs were summarized into themes by one investigator, and the frequencies of each theme were recorded. Those themes included: medical issues; skin integrity; equipment or supply needs; disruption to patient or family employment; mental health concerns; and ability to afford rent, food, and medications. Themes were then confirmed independently by two separate investigators as an effort to reduce bias.

## Results

Out of 89 previously served patients that were contacted, 51 answered and completed the needs assessment. 21 patients completed the needs assessment multiple months in a row because they had ongoing needs. Demographic information is summarized in Table 1. The majority of the patients were male and identified Spanish as their primary language. The most common diagnosis was stroke followed by spinal cord injury (Table 1).

**Table 1:** Demographics.

Age		
Age Range	21–77	
**Sex assigned at birth**	**N**	**%**
Male	37	72.54
Female	14	27.45
**Primary Language**	**N**	**%**
Spanish	35	68.62
English	14	27.45
Other	2	3.92156863
**Primary Diagnosis**	**N**	**%**
Stroke	23	45.09
SCI	10	19.60
TBI	7	13.72
Amputation	7	13.72
Other	4	7.84

Overall, the top three reported needs centered around themes of medical issues, equipment needs, and mental health concerns (Table 2). From the compiled reported concerns throughout the duration of the study, medical issues were reported with the highest frequency (46%), followed by equipment needs (30%) and mental health concerns (30%).

**Table 2:** Patient reported needs during COVID-19 pandemic.

	April	May	June	July	August	September	October	November	Total Number of Concerns (% Total)
Medical Issue	1	12	8	6	4	7	3	5	46 (15.18%)
Equipment	1	6	6	3	3	2	3	6	30 (9.90%)
Mental Health	1	10	6	5	2	2	1	3	30 (9.90%)
Rent	5	11	7	2	1	1	0	0	27 (8.91%)
Food	4	9	8	0	2	1	0	1	25 (8.25%)
Mobility/Functional Ability/ADLS	1	7	4	2	2	4	2	2	24 (7.92%)
Employment	2	12	9	0	0	0	0	0	23 (7.59%)
Cost of Medications	2	9	7	1	0	3	0	0	22 (7.26%)
Supplies	2	5	4	6	2	2	0	1	22 (7.26%)
Wound	0	4	1	1	2	2	1	3	14 (4.62%)
Medication Refills	1	2	2	1	2	1	0	0	9 (2.97%)
Transportation	0	0	5	0	1	1	0	2	9 (2.97%)
No Needs	0	8	5	4	1	2	1	1	22 (7.26%)

### Medical needs

Patients described unmet medical needs that went unaddressed due to a lack of access to healthcare. Some of the medical concerns were uncontrolled pain, pressure injuries or wounds, and concern for infection. In addition, patients reported an inability to obtain medication refills or cited prohibitive cost of their medications. Some patients also described lack of access to transportation to medical appointments.

### Equipment

Patients also described difficulties with equipment procurement and repairs. These patients experienced mobility challenges due to an inability to have their equipment fixed, adjusted, or replaced.

### Mental health

Patients frequently reported feelings of isolation.

Though less frequently reported, patients in this cohort also developed wounds, needed prescription refills, ran out of supplies, did not have accessible transportation, and expressed concern about their source of income and ability to afford basic necessities. Access to essential healthcare and life needs were disrupted.

Patient needs changed over time. Rent and employment concerns were more frequently expressed during early months, and equipment problems were more frequent in later months of the survey. A minority of contacted patients reported they had no needs, some of whom had acquired insurance.

## Discussion

During the early months of the COVID-19 pandemic, medical, equipment, and mental health concerns were the most frequent needs reported by our equity-deserving patients served by our pro bono interdisciplinary rehabilitation clinic. This is similar to other findings about individuals with physical disabilities, including prior reports that identified medical and equipment needs as primary concerns during the COVID-19 pandemic (4, 6). Previous studies also found mental health concerns were a significant issue for individuals with disabilities (3, 4).

The cohort of equity-deserving patients in this study also regularly expressed concerns related to SDoH. For example, patients discussed food insecurity, housing insecurity, and lack of transportation. It is well known that disparities in SDoH can lead to worse health outcomes. Disparate care exists for those with a disability defined by a spinal cord injury, stroke, amputation, or brain injury (3, 5). Medical care and resource allocation is vastly different for those with insurance and those without (8, 9). Those uninsured, equity-deserving patients who have sustained a catastrophic injury have limited access to healthcare, including rehabilitation services, durable medical equipment, and medications. It is the authors' conjecture, through lived experience at the pro bono interdisciplinary rehabilitation clinic, that this disparity was magnified by the COVID-19 pandemic.

### Limitations

There are limitations to our needs assessment. Only a fraction of patients could be reached to complete the needs assessment (ranging from 36%–64% of patients contacted each month). This occurred for a variety of reasons, including disconnected phone numbers, inaccurate contact information, or patients who opted not to answer their phone. It is possible that the patients who did not complete the assessment had different needs. In addition, this analysis was not a formal research study where empirical evidence was obtained or causation can be inferred. This needs assessment was conducted at one site, which limits the generalizability of our results.

Despite these limitations, this manuscript focuses on a group of patients that is rarely discussed and can guide future studies. It remains important to understand common needs from the beginning months of the pandemic, and further data collection could assess for potential changes in needs and SDoH over time through the COVID-19 pandemic. In addition, future research should involve prospective data collection to further illuminate the impact of emergencies on access to care. These data could provide a foundation for advocacy and planning, because policy changes are needed to increase access to equipment, supplies, and medical care for this equity-deserving population during times of crisis.

## Conclusion

Our objective was to identify and describe the needs of a racially and ethnically diverse set of uninsured individuals with physical disabilities seen at a specialized interdisciplinary rehabilitation pro bono clinic. Our manuscript provides information on high-priority needs for this cohort of underserved, equity-deserving patients during the early stages of the COVID-19 pandemic. Medical concerns, broken or unusable equipment, and mental health challenges were most frequently discussed. In addition, lack of transportation, inability to afford supplies or medication, as well as housing, food or job insecurity weighed heavily on our patients. Our healthcare system needs to understand and address these SDoH to provide appropriate support to these patients, especially if future public health crises or lockdowns occur.

## Data Availability

The raw data supporting the conclusions of this article will be made available by the authors, without undue reservation.
